# Abscess in the Testicle

**Published:** 1866-09

**Authors:** M. M. Eaton

**Affiliations:** Peoria, Ill.


### ABSCESS IN THE TESTICLE.

By M. M. EATON, M.D., of Peoria, Ill.

The extreme rarity of cases of this nature, causes me to place this one on record. Not that I have any new theories to offer, or treatment to suggest, but simply to aid in adding to the great storehouse of *medical* experience, by reporting such cases of interest as may be read by young members of the profession with profit. The history of this case is briefly as follows, *viz.:*—

A young man, aged 16 years, son of a merchant of this city, was placed under my care, June 4th, 1866. Found him complaining of great pain in the right iliac region, and on examination I discovered a tumor about the size of a large orange in this locality, very tender to the touch; and, on further investigation, I found the right testicle absent from the scrotum, and on inquiry, learned that the young man had, while a youth, about 6 years of age, passed this testicle up into the abdomen; that he had passed the left one up also, but it came down again; the right, however, still remaining in the abdomen, without causing any inconvenience whatever up to about a week since, at which time a physician was consulted, who prescribed for ague, as the patient had complained of a chill and had some fever, the doctor not being aware of any trouble in the abdomen or testicle, as the patient had carefully concealed the result of his foolishness in his early youth, so that even his parents were not aware of his condition. He grew rapidly worse, however, and on the 4th, I was requested to take charge of the case.

I found him with a wiry pulse, 110 to the minute; tongue with a white coat; skin dry; respiration 20 per minute. I applied fomentations of hops over the lower part of the abdomen; gave anodynes, diaphoretics, and diuretics, in connection with powders, every three hours, of hydrg. chlo. mit. gr. ij, opii pulv. gr. j. M.

June 5th. Seemed much improved. Pulse 100, and softer; skin moist; tenderness of abdomen less; suffering but little pain. The bowels not having moved, I ordered pill comp. cathar. No. ij, every four hours.

June 6th. No movement of bowels; pulse more wiry; commenced vomiting. Ordered injections of warm water, and continued diaphoretic mixture, and gave pill opii, gr. ij, every four hours, with ipecac in solution, $\frac{1}{12}$ gr. doses, to arrest the emesis and control the severe pain.

June 7th. Patient worse. No operation from several enemata that had been used. Tenderness over the whole abdomen; emesis continually, consisting of some fœcal matter, with water; complains of great thirst—the water drank is immediately rejected. Applied sinapism over the epigastrium, and gave plumb. acet. in solution, to arrest the vomiting.

June 8th. Patient worse every way. Passed air from the bladder with the urine. Used stimulating enemata with no success.

June 9th. Patient died. I requested a *post mortem* examination, in order to ascertain what condition the testicle really was in. My request was granted, and 12 hours after death I made the autopsy, assisted by Drs. J. Murphy and J. Hamilton, also, Drs. Hiatt and Potts.

We found, on opening the abdomen, large quantities of pus diffused throughout the entire peritoneal cavity, and on examining the testicle, which was retained in the abdomen for so long a time, enlarged from inflammation to about ten times its normal size, containing a cavity nearly as large as itself, which was open one side, through which the pus found in the peritoneal cavity had evidently escaped. The bladder was in a healthy condition; the kidneys normal; the liver slightly enlarged; the spleen healthy; the peritoneum intensely congested and softened; the duodenum softened through its muscular coat, so that it was torn by very slight violence; extensive evidence of inflammation throughout the entire tract of the alimentary canal, but no obstruction existed. The testicle was not removed, which I regret, as it would have been an interesting pathological specimen.

The bladder was inflated after drawing off two ounces of healthy urine, and found to be impervious. How shall we account for the gas which had been expelled through the urethra previous to death?

I am inclined to believe that, as the urine and bladder were in a normal condition, the gas must have formed in the inflamed testicle and passed through the spermatic duct into the urethra, and from thence expelled with the urine.

Another question may be asked:—Why did this retained testicle take on inflammatory action at this time, after having remained so long in its unnatural situation without producing any effect whatever on our patient?

I answer, that, in my opinion, the expulsion of the seminal fluid was prevented by the peculiar situation of the testicle, and that, in consequence, this fluid being retained in the testicle, it became an irritant, a foreign material causing the inflammatory action in the testicle and the resulting abscess, and the reason why this state of affairs had not long ago existed is found in that our patient had only recently arrived at the age of puberty, and, consequently, until recently, no seminal fluid had been secreted.

I do not wish to be understood as saying that where a testicle is retained in the abdomen until adult age, inflammation of the testicle is a certain result; for I know of a gentleman, at the present time in this city, the father of a fine, heaithy family, who has one of his testicles retained at the internal abdominal ring, and it has never troubled him; but that, where, from any cause, the spermatic fluid is prevented from leaving the testicle, it must necessarily bring on inflammatory action.

This case may also be instructive, in that the symptoms during the last three days of our patient's life were such as are described where some obstruction exists in the course of the alimentary canal; so much so, that the case was diagnosed by a very eminent and experienced physician, who saw the case with me on the day he died, to be one of invagination of the intestine, or, certainly, one of obstruction from cause. But, as I have stated, nothing of the kind was found to exist, although very diligent search was instituted.

